# Orderly display of limb lead ECGs raises Chinese intern’s diagnostic accuracy when determining frontal plane QRS axis

**DOI:** 10.1080/10872981.2018.1549923

**Published:** 2018-11-27

**Authors:** Gang Li, Kheshav Banarsee, Jari A. Laukkanen, Lan Hao

**Affiliations:** a Division of Cardiology, Department of Geriatrics, The First Affiliated Hospital of Chongqing Medical University, Chongqing, China; b Institute of Public Health and Clinical Nutrition, University of Eastern Finland, Kuopio, Finland; c Faculty of Sport and Health Sciences, University of Jyväskylä, Jyväskylä, Finland; d Department of Medicine, Central Finland Health Care District, Jyväskylä, Finland; e Chongqing Key Laboratory of Ultrasound Molecular Imaging, Institute of Ultrasound Imaging, Chongqing Medical University, Chongqing, China

**Keywords:** Electrocardiography, electrocardiogram, medical learning, medical teaching, medical education

## Abstract

**Background**: There is limited information on whether the orderly display of limb lead ECGs (electrocardiograms) can facilitate students to determine frontal plane QRS complex wave electrical axis.

**Objectives**: The study investigated whether the orderly display of limb lead ECGs can raise Chinese undergraduate intern’s diagnostic accuracy when determining frontal plane axis.

**Design**: A total of 147 fifth-year undergraduate interns aged between 21 and 25 years were randomly arranged into 2 groups: one group was given classically displayed ECGs of limb leads while the other group was given orderly displayed ECGs of limb leads. They were then taught to determine frontal plane axis with one of the above displays. The intern’s diagnostic accuracy and time used were measured.

**Results**: After teaching, the orderly display can more effectively raise diagnostic accuracy when determining axis as compared to the classical display (76.65 ± 23.16% vs. 68.88 ± 23.21%, *P* < 0.05), although diagnostic accuracy in axis determination was improved in both groups as compared to the axis determination at baseline (all *P* < 0.05).

**Conclusions**: Orderly display of limb lead ECGs may raise Chinese intern’s diagnostic accuracy when determining frontal plane axis.

## Introduction

**Figure 1.**
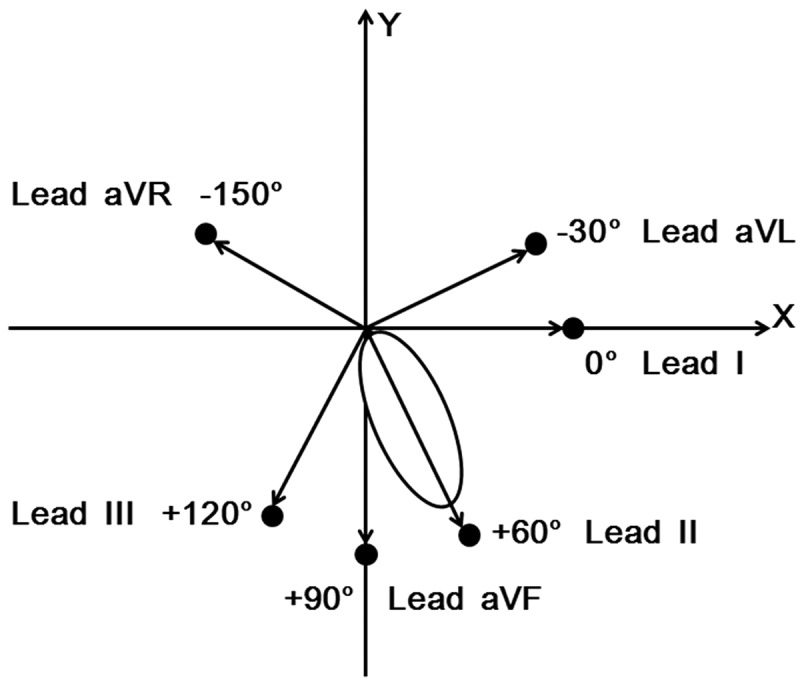
Schematic diagram of limb lead locations anatomically. 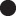, Anatomical location of limb leads; 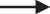, direction of cardiac electrical force sum vector received by limb leads; 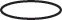, heart.

**Figure 2.**
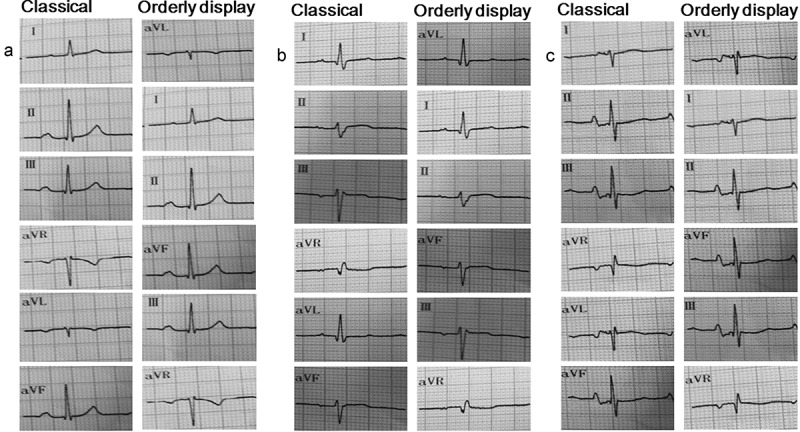
Electrocardiograms of limb leads in classical display and orderly display. (a) Note: representative electrocardiograms of normal axis. (b) Note: representative electrocardiograms of left-deviated axis. (c) Note: representative electrocardiograms of right-deviated axis.

Electrocardiogram (ECG) is the key and difficult point for medical students and staffs to learn perpetually. Frontal plane QRS complex wave electrical axis indicates the average direction of overall QRS on ECGs of six limb leads and reflects the main direction of ventricular electrical depolarization on frontal plane (Figure 1). Although frontal plane QRS electrical axis is a basic concept in electrocardiography, determination of frontal electrical axis is usually needed to the ECG definition of left anterior/posterior hemiblock, ventricular hypertrophy, and cor pulmonale [1,2]. In printout report, limb lead ECGs are displayed classically in the order when they were invented (I, II, III, aVR, aVL, aVF, Figure 2) rather than according to their anatomically orderly locations (aVL, I, II, aVF, III, aVR, Figure 2). The classical display of limb lead ECGs has brought great difficulties for medical students to learn limb lead ECGs interpretation and to identify frontal plane QRS electrical axis of limb lead ECGs, because there is no regulation to follow the evolution of limb lead ECGs according to the classical display of limb lead ECGs. After aVR is inverted into −aVR by automated digital ECG analysis system [3,4], all six limb lead ECGs can be presented orderly in a single Cabrera’s sequence (aVL, I, −aVR, II, aVF, III) [5–7]. The anatomically orderly display of limb lead ECGs can facilitate to view the sequential progression of cardiac electrical activity in frontal plane, especially for the students who have no previous experience with ECG interpretation. In recent years, studies have proved that Cabrera’s anatomically orderly display of limb lead ECGs is also helpful for the localization of myocardial infarction, acute stress injury, and arrhythmic origin [8–12]. Furthermore, a study has found that the use of Cabrera’s orderly display of limb lead ECGs (aVL, I, −aVR, II, aVF, III) could raise the student’s diagnostic accuracy in less amount of time than the classical display (I, II, III, aVR, aVL, aVF, Figure 2) when determining frontal plane QRS axis [13]. However, −aVR, a fictitious limb lead, has not been implemented in practice, which may have brought negative impact on students to analyze ECGs and frontal plane QRS electric axis. Therefore, we hypothesized that the use of anatomically orderly display of limb lead ECGs (aVL, I, II, aVF, III, aVR, Figure 2) can similarly elevate student’s diagnostic accuracy in lesser time than the classical display (I, II, III, aVR, aVL, aVF, Figure 2) when determining frontal plane QRS axis.

## Methods

### Study design

In this study, 147 fifth-year undergraduate medical students aged between 21 and 25 years (58 males, 89 females) were recruited from our university hospital. All of them had previous experiences with ECG interpretation. The participants were randomly arranged into classical ECG display and orderly ECG display groups, respectively, using digital random counter.

### Teaching method

Web-assisted and lecture-based tutorial were given to both groups [14]. Students in both groups received the same instruction about the cardiac basic anatomy, mechanics, and electrophysiology. The same instructor explained the anatomic positions of different leads and the formation mechanisms of normal ECGs and abnormal ECGs that resulted from various electrical axis deviations for each lead and outlined the steps required for axis calculation and visually defined the electrical axis. Four examples of ECGs were used for the instruction of determining electrical axis in each group. All ECG papers’ running speed in this study was 25 mm/s. Except for different display of ECGs, the example ECGs and the relevant teaching were same between classical and orderly display groups. In these two groups, each example ECG was from the same patient but in different display. The classical group used the classical limb lead display (I, II, III, aVR, aVL, aVF, Figure 2) when determining the electrical axis, while the orderly group used the anatomically orderly limb lead display (aVL, I, II, aVF, III, aVR, Figure 2).

### Evaluation methods

**Figure 3.**
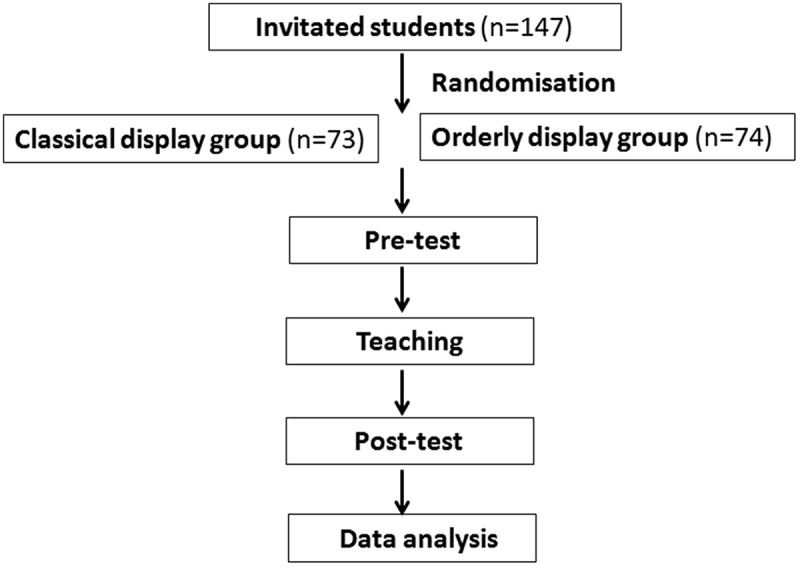
Schematic diagram of participant’s subgroups, teaching, pretest, posttest, and data analysis.

Except for different display of ECGs, test contents including number of question and the placement positions of each test ECG lead were same between classical and orderly display groups. In the two groups, each test ECG corresponded the same question number that was from the same patient but in different display. Students of both groups undertook two tests: a pretest (before teaching) and a posttest (1 week after teaching) (Figure 3). Each test contained the same contents using the same eight test ECGs but in different display for each group. Correct answer for each ECG scored one point. The question was to determine the electrical axis. All answers were recorded on an answer sheet on which the participants were asked to state their age and sex. The possible axis test scores ranged from 0 to 8, and it was expressed as a percentage. The test ECGs included four ECGs with normal electrical axis ranged from −30° to +90° (Figure 2(a)), two ECGs with left-deviated axis from −30° to −90° (Figure 2(b)) and two ECGs with right-deviated axis from +90° to +180° (Figure 2(c)). The investigators determined the correct electrical axis by consensus on all above ECGs. They were selected from a database of digitized ECGs at our university hospital. A single-answer question was set for each ECG with three possible options including normal electrical axis, left-deviation, and right-deviation. The time used to complete the answer sheet was measured by stopwatch and noted for each student.

The study protocol was conducted in accordance with the Declaration of Helsinki and approved by the institutional ethics committee. The participants were aware of the investigational nature of the study and agreed to participate after informed consent.

### Statistical analysis

Continuous parameters were expressed as mean values ± standard deviation. The statistical differences were evaluated by two independent sample's *t* test. Categorical data were compared using Chi-square test. Pearson univariate correlation was utilized for data analysis. Statistical analysis was performed by SPSS 22.0 software package (IBM Company, Chicago, Illinois 60606, USA). A two-tailed value of *P* < 0.05 was considered to be statistically significant.

## Results

There were no differences in participant’s age, sex, and baselines of diagnostic accuracy and time used in axis quiz before teaching between the classical and orderly display groups (*P* > 0.05, Table 1). After teaching, the diagnostic accuracy was higher in the orderly display group as compared to the classical display group (*P* < 0.05, Table 1); although the time used trended to become shorter after the teaching in the orderly display group, there was still no difference in time used in axis quiz between the two groups (*P* > 0.05, Table 1). However, after teaching, the diagnostic accuracy was more elevated in both groups as compared to their respective baseline (all *P* < 0.05, Table 1).

**Table 1.** Comparison of student’s characteristics, diagnostic accuracy, and time used in quizzes of cardiac frontal plane QRS axis between limb lead classical and orderly display groups.

	Classical display (*n* = 73)	Orderly display (*n* = 74)	*p* value
Age (year)	22.66 ± 0.71	22.73 ± 0.90	0.590
Sex (M/F, *n*)	30/43	28/46	0.689
Accuracy before teaching (%)	33.38 ± 26.76	36.00 ± 30.13	0.583
Time used before teaching (min)	4.53 ± 1.97	5.04 ± 2.21	0.144
Accuracy after teaching (%)	68.88 ± 23.21^†^	78.65 ± 23.16^†,^*	0.012
Time used after teaching (min)	5.01 ± 1.81	4.82 ± 2.16	0.577

Data are presented as mean value ± standard deviation. **^†^***p* < 0.05 versus before teaching; *p < 0.05 versus classical display group.

## Discussion

In the present study, after teaching, anatomically orderly display of limb lead ECGs (aVL, I, II, aVF, III, aVR, Figure 2) can more effectively increase Chinese undergraduate medical intern’s diagnostic accuracy when determining frontal plane QRS axis as compared to the classical display (I, II, III, aVR, aVL, aVF, Figure 2), although the diagnostic accuracy in the axis was higher in both groups as compared to their respective baseline.

Physicians from any specialty have to possess the basic skills of ECG interpretation and rapidly identify potentially life-threatening diseases such as acute myocardial infarction and malignant arrhythmias derived from an ECG tracing [15]. Incorrect ECG interpretation can lead to misdiagnosis and inappropriate treatment decisions, including fatal events among patients with suspected cardiac diseases. However, a lack of these basic ECG interpretation skills is still a serious concern among many physicians [15–17]. Survey revealed that a significant proportion of medical graduates were unable to correctly interpret ECGs [18]. The competency for ECG interpretation was associated with the adequacy of the graduating medical student’s ECG education and training [19]. One potential reason for the graduate’s inability to correctly interpret the ECGs may be a result of poor medical education. However, studies showed that the display of limb lead ECGs can also impact the skills of ECG interpretation. Cabrera’s anatomically orderly display of limb lead ECGs can increase student’s diagnostic accuracy in lesser time than the classical display when determining frontal plane QRS axis [13].

In the present study, Chinese undergraduate medical intern’s diagnostic accuracy reached 33.3–36.0%, respectively, when determining the frontal plane QRS axis in both groups before tutorial. These findings were similar with previous reports [18,19]. It confirmed that Chinese undergraduate medical interns were also short of the abilities to interpret the ECGs, although they have had previous experiences with ECG interpretation. The ECG instruction for Chinese undergraduate medical students still seems to be limited. There is necessity to increase Chinese medical student’s ability to correctly interpret ECG during undergraduate education via reforming methods of ECG teaching, training, and tests [20]. Due to appropriate teaching, orderly display of limb lead ECGs was confirmed to effectively raise Chinese medical intern’s diagnostic accuracy when determining frontal plane QRS axis. This finding was similar to Pahlm’s study [13]. Nevertheless, the orderly display sequence of limb lead ECGs used by us was aVL, I, II, aVF, III, aVR (Figure 2), which was really anatomical order, while Pahlm used Cabrera’s orderly display sequence of limb lead ECGs. The Cabrera’s sequence is aVL, I, −aVR, II, aVF, III, among which, −aVR is a fictitious limb lead [13]. It seems that orderly display of limb lead ECGs is convenient for students to observe the systematically organized evolution of cardiac electrical activity and ECG graphs. The orderly display of limb lead ECGs can facilitate students to analyze cardiac electrical axis and improve the diagnostic accuracy of ECG. However, our orderly display of limb lead ECGs has not been proved to shorten the time used by Chinese medical interns in determining frontal plane QRS axis, although similar observations were never documented in a previous study [13]. Moreover, the diagnostic accuracy in the electrical axis was improved in both groups based on a tutorial session. This positive teaching effect is considered to result from the combined use of lecture-based and internet-assisted teaching methods, which is consistent with previous findings [14,19]. These results also suggested that continued ECG education is needed to prevent undergraduate medical trainee’s decay of ECG knowledge and maintain their diagnostic accuracy and skills of ECG interpretation when determining frontal plane QRS axis.

Although classical display of limb lead ECGs (I, II, III, aVR, aVL, aVF, Figure 2) has many drawbacks, it is still used traditionally in majority countries. However, combined with the results of this study, the orderly display of limb lead ECGs (aVL, I, II, aVF, III, aVR, Figure 2) is recommended for use in the future clinical practice, because it provided better accuracy for the diagnosis of cardiac electric axis, as compared with the classical display. Based on the current study, our orderly display of the limb lead ECGs (aVL, I, II, aVF, III, aVR, Figure 2) could be more favored, as compared with the Cabrera’s orderly display (aVL, I, −aVR, II, aVF, III), because it is easily available to obtain and may help to set some important ECG-based diagnoses.

However, this study was carried out in a single center and did not assess the effects of the teaching on long-term retention of ECG interpretation skills. The findings remain to be reconfirmed in the future relevant trials.

## Conclusions

Orderly display of limb lead ECGs may raise Chinese undergraduate medical intern’s diagnostic accuracy when determining frontal plane QRS axis.

## References

[1] Obasohan AO, Egbagbe EE, Bazuaye AE. The electrical axis of the heart in nigerian patients with chronic obstructive lung disease in Benin City. West Afr J Med. 2011;30(4):288–5. 22669835

[2] Abreu-Lima C, Jp MDS, Coelho G, et al Frontal-plane QRS axis revisited: accuracy of current approximations and reappraisal of their merit in the diagnosis of right ventricular hypertrophy. J Electrocardiol. 1988;21(4):369–375. 2977149 10.1016/0022-0736(88)90114-8

[3] Fumagalli B Unipolar value of standard limb leads; lead -VR and rational arrangement of limn leads. Am Heart J. 1954;48(2):204–223. 13180473 10.1016/0002-8703(54)90174-x

[4] Case RB, Moss AJ Recommendation for revision of the standard presentation of the frontal plane ECG leads including reversal of lead aVR (to -aVR): it is time for a change. Ann Noninvasive Electrocardiol. 2010;15(2):97–100. 20522048 10.1111/j.1542-474X.2010.00348.x PMC6932651

[5] Anderson ST, Pahlm O, Selvester RH, et al Panoramic display of the orderly sequenced 12-lead ECG. J Electrocardiol. 1994;27(4):347–352. 7815015 10.1016/s0022-0736(05)80275-4

[6] Dower GE, Nazzal SB, Pahlm O, et al Limb leads of the electrocardiogram: sequencing revisited. Clin Cardiol. 1990;13(5):346–348. 2347126 10.1002/clc.4960130508

[7] Graettinger JS, Packard JM, Graybiel A A new method of equating and presenting bipolar and unipolar extremity leads of the electrocardiogram; advantages gained in visualization of their common relationship to the electric field of the heart. Am J Med. 1951;11(1):3–25. 14837921 10.1016/0002-9343(51)90003-4

[8] Kosuge M, Kimura K Implications of using the cabrera sequence for diagnosing acute coronary syndrome. Circ J. 2016;80(5):1087–1096. 27019984 10.1253/circj.CJ-16-0126

[9] Menown IB, Adgey AA Improving the ECG classification of inferior and lateral myocardial infarction by inversion of lead aVR. Heart. 2000;83(6):657–660. 10814623 10.1136/heart.83.6.657 PMC1760884

[10] Perron A, Lim T, Pahlm-Webb U, et al Maximal increase in sensitivity with minimal loss of specificity for diagnosis of acute coronary occlusion achieved by sequentially adding leads from the 24-lead electrocardiogram to the orderly sequenced 12-lead electrocardiogram. J Electrocardiol. 2007;40(6):463–469. 17993301 10.1016/j.jelectrocard.2007.07.002

[11] Lam A, Wagner GS, Pahlm O The classical versus the Cabrera presentation system for resting electrocardiography: impact on recognition and understanding of clinically important electrocardiographic changes. J Electrocardiol. 2015;48(4):476–482. 26051487 10.1016/j.jelectrocard.2015.05.011

[12] Sgarbossa EB, Barold SS, Pinski SL, et al Twelve-lead electrocardiogram: the advantages of an orderly frontal lead display including lead -aVR. J Electrocardiol. 2004;37(3):141–147. 15286926 10.1016/j.jelectrocard.2004.04.002

[13] Pahlm US, O’Brien JE, Pettersson J, et al Comparison of teaching the basic electrocardiographic concept of frontal plane QRS axis using the classical versus the orderly electrocardiogram limb lead displays. Am Heart J. 1997;134(6):1014–1018. 9424060 10.1016/s0002-8703(97)70020-6

[14] McClennen S, Nathanson LA, Safran C, et al ECG wave-maven: an internet-based electrocardiography self-assessment program for students and clinicians. Med Educ Online. 2003;8(1):4339. 28253171 10.3402/meo.v8i.4339

[15] Novotny T, Bond RR, Andrsova I, et al Data analysis of diagnostic accuracies in 12-lead electrocardiogram interpretation by junior medical fellows. J Electrocardiol. 2015;48(6):988–994. 26381796 10.1016/j.jelectrocard.2015.08.023

[16] Ochsmann EB, Zier U, Drexler H, et al Well prepared for work? Junior doctors’ self-assessment after medical education. BMC Med Educ. 2011;11:99. 22114989 10.1186/1472-6920-11-99 PMC3267657

[17] de Jager J, Wallis L, Maritz D ECG interpretation skills of South African emergency medicine residents. Int J Emerg Med. 2010;3(4):309–314. 21373298 10.1007/s12245-010-0227-3 PMC3047864

[18] Lever NA, Larsen PD, Dawes M, et al Are our medical graduates in New Zealand safe and accurate in ECG interpretation? N Z Med J. 2009;122(1292):9–15. 19448769

[19] Jablonover RS, Lundberg E, Zhang Y, et al Competency in electrocardiogram interpretation among graduating medical students. Teach Learn Med. 2014;26(3):279–284. 25010240 10.1080/10401334.2014.918882

[20] Raupach T, Harendza S, Anders S, et al How can we improve teaching of ECG interpretation skills? Findings from a prospective randomised trial. J Electrocardiol. 2016;49(1):7–12. 26615874 10.1016/j.jelectrocard.2015.10.004

